# A Maximum Entropy Species Distribution Model to Estimate the Distribution of Bushpigs on Madagascar and Its Implications for African Swine Fever

**DOI:** 10.1155/2023/7976252

**Published:** 2023-02-28

**Authors:** José Manuel Díaz-Cao, Nárjara Grossmann, Steven M. Goodman, Jaime Bosch, Helene Guis, Miatrana Rasamoelina, Rianja Rakotoarivony, Ferran Jori, Beatriz Martínez-López

**Affiliations:** ^1^Center for Animal Disease Modeling and Surveillance, Department of Medicine and Epidemiology, School of Veterinary Medicine, University of California Davis, Davis, USA; ^2^Departamento de Patoloxía Animal, Facultade de Veterinaria Lugo, Universidade de Santiago de Compostela, Lugo, Spain; ^3^Field Museum of Natural History, Chicago, USA; ^4^Association Vahatra, Antananarivo, Madagascar; ^5^Animal Health Department, Centro de Vigilancia Sanitaria Veterinaria (VISAVET), Complutense University of Madrid, Madrid, Spain; ^6^Epidemiology and Clinical Research Unit, Institut Pasteur de Madagascar, Antananarivo, Madagascar; ^7^ASTRE, Université Montpellier, CIRAD, INRAE, Montpellier, France; ^8^FOFIFA-DRZVP, Antananarivo, Madagascar; ^9^Department of Zoology and Entomology, University of Pretoria, Pretoria, South Africa

## Abstract

Bushpigs (*Potamochoerus larvatus*) play a major role in the socio-ecosystem of Madagascar, particularly in rural areas. They are largely hunted by rural populations as a major source of income and protein. They can also represent a potential source of pathogens for domestic animals and people. For example, it is hypothesized that bushpigs might compromise African swine fever (ASF) eradication programs by sporadically transmitting the virus to domestic pigs. However, available knowledge on the distribution of bushpigs in Madagascar is limited. In this study, we estimated the distribution of bushpigs on Madagascar using a species distribution model (SDM). We retrieved 206 sightings of bushpigs in Madagascar during 1990–2016 and predicted the distribution by using 37 climatic, geographic, and agricultural/human variables related to the presence of bushpigs and running a presence-background maximum entropy SDM. Our model identified three main areas with a high suitability for bushpigs: in the north, central-western, and east of the island (AUC = 0.84). The main contributors to the model were the vegetation index (51.3%), percentage of land covered by trees (17.6%), and annual averaged monthly precipitation (12.6%). In addition, we identified areas in central Madagascar with a high density of domestic pigs and a high suitability score for bushpigs. These results may help to identify bushpig areas at the interface with domestic pigs to assess the risk of pathogen transmission and to design ecological assessments, wildlife management studies, or targeted surveillance and research studies related to many bushpig-borne pathogens, such as ASF, which is an endemic problem in the country, as well as zoonotic diseases such as cysticercosis and hepatitis E. Our approach could also be extrapolated to other species of wild swine in other countries.

## 1. Introduction

Wild pigs have been shown to carry a large diversity of pathogens in many parts of the world, many of which can induce disease in other domestic animals and humans [1]. In Madagascar, the bushpig (*Potamochoerus larvatus*) represents the largest terrestrial vertebrate. This species is not native and is suspected to have been introduced in the country more than 2000 years ago, with the arrival of the first human populations from the African continent [2]. Bushpigs are widespread in the Malagasy territory and represent an important source of income and protein for many rural populations in the country, but they are also important crop raiders [3]. As such, they have the capacity to approach anthropized areas and interact with domestic pigs, and, therefore, we hypothesize a considerable risk of direct or indirect pathogen transmission to pigs and other domestic animals. The role of bushpigs as source of pathogens in the African continent and Madagascar has been insufficiently studied [4]. However, there is occasional evidence of their capacity of carrying pathogens related to important diseases such as bovine tuberculosis [5], trichinellosis [6], classical swine fever [7], and African swine fever (ASF) [8].

To properly quantify this risk, it is necessary to determine the distribution of bushpigs and the potential areas of contact with domestic pigs. This will be useful to understand the epidemiology of shared diseases in the country such as ASF. The ASF is one of the major transboundary diseases in swine due to its health and socioeconomic impact in the affected countries. In Madagascar, this disease is endemic despite long efforts to eradicate it after its introduction in 1997 [9, 10]. Cycles involving wild suids, ticks, and domestic pigs contribute to the maintenance of the disease in endemic countries of Africa [11], but it is uncertain whether they occur in Madagascar. However, the potential of this swine to interact and share ASF virus, as well as other pathogens, with domestic pigs in Madagascar remains unstudied.

However, data on the presence of bushpigs in Madagascar are scarce and scattered. Species distribution models (SDMs) of maximum entropy (MaxEnt) are widely used tools that have successfully helped to estimate the suitability of areas for the presence of several species based on environmental, climatic, and anthropogenic factors [12]. In this sense, efforts to characterize the wild boar-domestic pig interface are carried out by the ENETWILD consortium in Europe with similar approaches to define risk maps of interactions [13, 14], but these initiatives are more limited in African countries, even though important shared diseases are endemic. Thus, in this study, we aimed to apply an SDM to estimate the distribution of bushpigs in Madagascar using the MaxEnt algorithm. This would help to define areas in which contact between domestic and wild pigs might be likely and provide the foundations to conduct further epidemiological studies at the bushpig-domestic pig interface and risk-based surveillance.

## 2. Material and Methods

### 2.1. Study Area

Madagascar can be divided into three main geographic areas: (i) the eastern coast, with a narrow and steep escarpment characterized by lowland moist evergreen forests (sea-level to about 900 m); (ii) the central highlands (the zone above 900 m), which comprises the most densely populated part of the island and concentrates the rice-producing lands between patches of highly fragmented medium-altitude moist evergreen forest; and (iii) a western and southern area characterized by dry deciduous forests, dry spiny thickets, and secondary grasslands and pastures (vegetation formations follow Gautier et al. [15]).

### 2.2. Data on Bushpig Presence

We obtained georeferenced data using the waypoint WGS84 on the presence and occurrence of bushpigs obtained during broad-scale biological inventories of mammals made by the Vahatra Association during the period 1990–2016 (*n* = 206). The data collected for this study are the result of multiple projects to assess the presence and distribution of animals on the island [16]. This consisted of visits to register observations of bushpigs and their tracks and spoor, as well as remains from hunter camps. Indirect evidence of bushpigs was considered, as this is the only wild swine species present in the country, but it was also discussed with local people.

In the SDM, problems of pseudoreplication may occur due to neighboring observations that may present similar values for environmental variables, violating the assumption of independence between observations and compromising the reliability of the model [17]. Therefore, to control for potential pseudoreplication problems, we reduced the local density of occurrences by removing observations under a determined pairwise distance [18]. This threshold was established at 15 km as bushpigs in Africa have been reported to move to a maximum distance of 15 km [19]. We intended with this that the observations represent different suitable habitats for bushpigs. As a result, the number of observations of bushpigs in the period of study used was reduced from 206 to 83 (Supplementary Figure 1). When various observations were closer than the threshold, one of them was randomly selected for modeling.

### 2.3. Explanatory Variables

We conducted a comprehensive review of the literature to identify possible factors that influence the presence and distribution of bushpigs [20–24]. We selected 37 variables and grouped them into four categories: climatic, vegetation, geographic, and agricultural/human. The variables and their sources are detailed in Supplementary Table 1. Briefly, they are defined as follows:

  Climatic variables were obtained from WorldClim 2 (BIOCLIM, [25]). These data consist of averages for the years 1970–2000 from climatic variables interpolated from ground-based meteorological measurements. These consisted of monthly/annual rasters for temperature (mean, minimum, and maximum), annual-averaged monthly precipitation, wind speed, solar radiation, and water vapor pressure. In addition, we used 17 other bioclimatic variables available from WorldClim 2 that represent annual trends, seasonality, and extreme or limiting environmental factors.
  Vegetation variables consisted of the normalized difference vegetation index (NDVI), evapotranspiration, and the percentage of land area covered by trees, nontree vegetation, and no vegetation. These variables are proportional estimates of cover developed from global training data derived using high-resolution imagery and were obtained from the moderate resolution imaging spectroradiometer (MODIS) aboard NASA's Terra satellite and extracted using the R package “MODIStsp” [26]. All rasters available for the period of study in the MODIS (from February 2000 to December 2016; no data prior to February 2000 was available in this source) were gathered. These consisted of 204 monthly rasters for NDVI and a raster from 2016 for the percentage of land area covered by trees, nontree vegetation, and no vegetation. We used the NDVI rasters to calculate the NDVI seasonality as the coefficient of variation of the average monthly rasters. The procedure to obtain a single raster of the period consisted of averaging submonthly rasters to monthly rasters, then to annual rasters, and finally to a raster of the entire period.
  Geographic variables consisted of elevation retrieved from WorldClim 2 BIOCLIM [25], distance to water points (rivers, wetlands, and lakes), and distance to the nearest main human settlements (cities and towns). This refers to human settlements with more than 1,000 inhabitants. Layers of water points and human settlements were obtained from Open Street Maps [27] and were used to create rasters of the Euclidean distances.
  Agricultural/human variables consisted of raster maps of the production of Madagascar's principal crops (rice and cassava). These data were retrieved from the International Food Policy Research Institute [28]. These datasets were created by spatially disaggregating national and subnational harvest data for 2001 using the Spatial Production Allocation Model (SPAM). This approach is a cross-entropy method that estimates crop distribution from a variety of inputs (land cover, crop production statistics, biophysical suitability, rural population density, etc.) [29, 30]. In addition, a raster of the human population in Madagascar (number of people per pixel) was obtained from WorldPop [31], which uses a dasymetric modeling approach for calculation. Finally, a raster of the human footprint was retrieved from the Socioeconomic Data and Applications Center of NASA [32], and it is defined as the cumulative human pressure on the environment and calculated as a combination of eight variables: built-up environments (human produced areas that provide the setting for human activity, e.g., buildings, paved land, and urban parks), population density, electric power infrastructure, crop lands, pasture lands, roads, railways, and navigable waterways [32].

All predictor rasters were standardized to the same extent and projection (WGS 84/UTM zone 38). The cell sizes of the different rasters were set following the layer with the lowest resolution (926 × 926 m).

### 2.4. Species Distribution Model

The suitability for the presence of bushpigs on Madagascar was calculated using an SDM based on the MaxEnt algorithm [33]. MaxEnt was developed to use presence-only data by contrasting presences with background locations [12] and has been shown to outperform other algorithms, even when applied to small datasets [34, 35]. In MaxEnt, it is essential to place background points where presence/absence is unmeasured but in which the presence is possible. Thus, a random sample of 10,000 background points [36] was obtained from the environmental layers after removing locations which were not expected to be permanent habitats for these animals (water points, roads, trail roads, and human settlements, as defined above). Since observations of animals may be easier in areas with more human presence, we used the human footprint layer as a bias layer to distribute 10,000 background points with a likelihood of presence proportional to this layer [37].

We ran separate MaxEnt models for each of the four groups of variables (i.e., climatic, vegetation, geographic, and agricultural/human). In each of the four models, we evaluated collinearity following a data-driven variable selection approach. First, we computed the pairwise Spearman's correlation of each variable and set a threshold of 0.7 [38]. Then, we performed a leave-one-out Jackknife test among all correlated variables, thus ruling out the correlated variable that least decreased the model's performance. This performance was measured with the Akaike's Information Criterion corrected for a small sample size (AICc). We also used the area under the curve (AUC) of the receiver operating characteristic (ROC) and the true skill statistic (TSS) to explore if using different metrics would lead to different results. The process was repeated after removing each variable until the correlations among all the retained variables fell below the threshold. Finally, a dataset that included all the selected variables from each of the four groups was run, repeating the same collinearity assessment. Spearman's correlations between all the variables are shown in Supplementary Figure 2.

We tuned the model by selecting the regularization multiplier, the features to be used, the number of iterations, and the inclusion/exclusion of explanatory variables, removing first the variable with the lowest contribution to the model as suggested by Phillips et al. [33] according to the best AICc value. To determine the optimal model complexity, we explored all combinations of the regularization parameter from 0.1 to 6 at intervals of 0.2 and nine potential combinations of four feature classes: linear “*l*,” quadratic “*q*,” product “*p*,” and hinge “*h*” (“*l*,” “*lq*,” “*lp*,” “*lqp*,” “*h*,” “*lh*,” ‘*lqh*,” “*lph*,” “*lqph*”) and set a final model with 100 replications. Since the predictive accuracy of models selected by AICc has been questioned [39], we also used AUC and TSS to explore if using different metrics would produce a different solution.

The models' performances were evaluated by cross validation by splitting our dataset into four spatially independent partitions. We used three partitions to train the model and the remaining one to test it. The accuracy of the final model was estimated by computing the AUC and the partial ROC (pROC) [40]. We verified the absence of spatial sorting bias using the function ssb in the R package “dismo” [41]. The models, the selection of variables, and the model tuning were carried out using the R package “SDMtune” [42].

The variables in the final model were ranked based on the estimated percentage contribution. The resulting model was expressed on a map (resolution: 926 km × 926 km) using the maximum value (pointwise) of the 100 replications to provide the scenario of highest suitability. The model's predictions are given in logistic format and represent a suitability score (0-1) of the cell for the presence of bushpigs. All the values presented were obtained as averages of the cross-validation runs. We also estimated the most limiting variables (responsible for decreasing suitability) in each raster cell using the function limiting implemented in the R package “rmaxent.” This function defines, for each cell, the variable whose value is most responsible for decreasing suitability in this model [43, 44]. In addition, we explored the influence on the output of resampling the presence points to avoid pseudoreplication by repeating the models 1,000 times, each time with a different random set of presence points and ensuring again a minimum distance of 15 km between points, creating some functions in R to automatize the process of point selection and subsequent analysis (Supplementary Code 1).

In order to detect areas with high risk of contact between domestic pigs and bushpigs, we collected data on the distribution of the density of domestic pigs in Madagascar in 2006 available from the livestock geo-wiki (a multipartner collaboration of the International Livestock Research Institute, the Food and Agriculture Organization of the United Nations, and the Université Libre de Bruxelles) and calculated by Gilbert et al. [45] (Supplementary Figure 3), and we compared the livestock density map of Madagascar with the results of our model. For this purpose, we selected the pixels with the highest quartile of the density of domestic pigs and the highest suitability score for bushpigs estimated by our model and compared the areas in which both maps overlap.

## 3. Results

**Figure 1:**
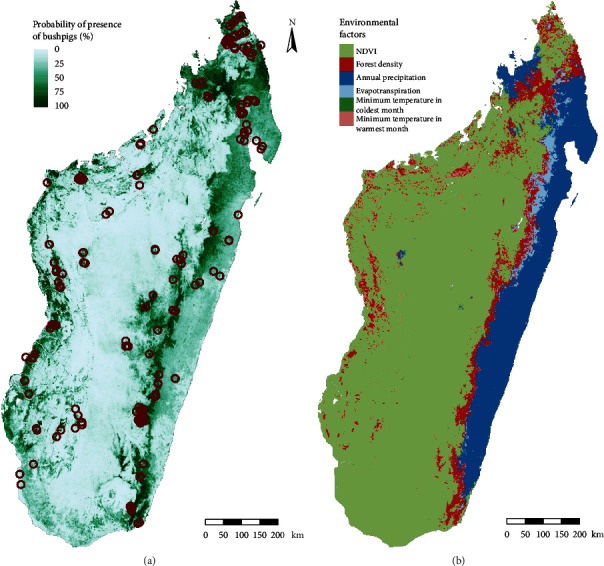
(a) The map of the estimated probability of presence of bushpigs on Madagascar obtained with the species distribution model and presence data (*n* = 206), and (b) the map of the limiting factors of the environmental suitability for bushpigs predicted by the model.

The best model according to AICc included linear and quadratic features and a regularization parameter of 0.4. Using different metrics (AUC or TSS) to assess model selection did not change the final selected model in this case. Model performance was notably good with a high AUC value (0.84; sd = 0.006) and a pROC of 0.84 (*p* < 0.001). The suitability for bushpigs predicted by the model is shown in Figure 1(a). The resampling of the presence points did not show great variation in the output since in all the iterations the variation of the suitability scored in each cell was lower than 5% in 95% of the raster cells (Supplementary Figure 4) compared to the presented model.

**Figure 2:**
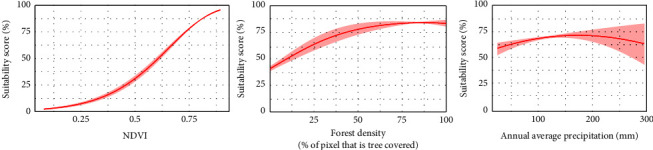
Response curves of the three main contributors of the ecological niche model.

The final model included six variables (Table 1) after removing those that displayed collinearity and low contribution to the model. The main contributors to our model were NDVI (51.3%), percentage of land covered by trees (17.6%), and annual average monthly precipitation (12.6%) (Table 1).  Figure 1(b) shows that NDVI is the most common predictor associated with the largest decrease in suitability in each pixel. For these three principal contributors, the response curves showed that the probability of the presence of bushpigs increased with variables related to vegetation cover and precipitation (Figure 2).

**Figure 3:**
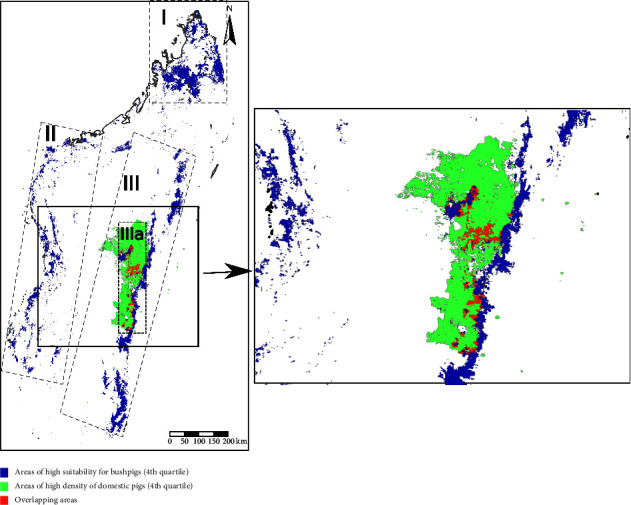
The map of the areas of high density of domestic pigs and probability of presence of bushpigs and overlapping areas between both species. Three zones (I, II, and III) of high probability of bushpigs are differentiated and a subzone where overlapping distribution with domestic pigs is more likely (IIIa).

The geographic areas that presented a probability greater than 75% (highest quartile) of the presence of bushpigs were mainly concentrated in the north of the island, in the central west, and in the eastern area including a portion of the central highlands (zones I, II, and III in Figure 3, respectively). The density distribution of the domestic pigs presented few high values, and 75% of the pixels presented less than 10 heads per km^2^. The high-density areas of domestic pigs were mainly located in the central highlands (Supplementary Figure 2). Comparing the areas of higher probability of bushpigs (>75%) with the areas of higher density of domestic pigs (>10 heads/km^2^), we found several overlapping pixels in the central highlands and eastern portions of Madagascar (zone IIIa in Figure 3).

## 4. Discussion

Ecological and health studies on African forest wild pig species such as *Potamochoerus porcus* and *Hylochoerus meinertzhageni* are scarce and scattered, mainly due to the elusive nature and nocturnal habits of these species. The bushpig is not an exception, and knowledge about its precise area of distribution and its potential role as a source of pathogens on Madagascar is still limited. Consequently, in this study, we estimated the distribution of bushpigs on Madagascar using the SDM. The approach followed in the present study also maximized the use of information from open-source databases and allowed us to provide, to the best of our knowledge, the first suitability map for this species on Madagascar. Moreover, this approach also allows a quantification for this suitability, so that surveillance can be designed considering the certainty about the presence of bushpigs at each particular location and efforts can be weighted accordingly.

Defining the area of bushpig distribution is a key element to realistically assess the risk of contact with domestic pigs and its role in interspecies disease transmission. According to previous studies, bushpigs occur throughout much of sub-Saharan Africa in a variety of wooded and wetland habitats including closed canopy moist forest [8, 46]. On Madagascar, the range of this species was previously estimated to be lowland areas, forming a sort of ring around the island and not occurring in the central highlands [8]. The bushpig distribution on Madagascar estimated by our model is largely consistent with this previous approximation but add greater resolution, showing a pattern of distribution in the central highlands and determining specific areas of high probability for its occurrence, particularly in the north, in the central-western and eastern half of the island. Our analysis provides new insights into the areas of distributional overlap in eastern half of the island, particularly the central highlands (zone IIIa in Figure 3) between bushpigs and domestic pigs, which may favor interspecies transmission of pathogens.

The results of our model on the distribution of bushpigs on Madagascar were mainly determined by variables related to vegetation: the NDVI and the percentage of land covered by trees. The NDVI is a measure of the presence of live green vegetation associated with near-infrared sunlight reflected by the plant canopies. Different studies have shown that several ungulate populations in Africa react to variation in the NDVI with regards to their behavior, distribution, etc. (e.g., [47–50]). The high contribution of the NDVI to the model (51.3%) also points to the utility of this parameter as a screening of the potential presence of bushpigs. Diverse studies have shown a preference for bushpigs in areas with dense vegetation [23, 24], which is consistent with the relationship in our model with those areas with higher tree coverage. Nevertheless, although the NDVI and forest density are variables that may be interrelated, the correlation between them was below 0.7, and the greater contribution of the NDVI suggests that the presence of bushpigs is not only restricted to high-density forests but that animals may inhabit areas with different vegetation covers as evidenced by the presence of observations in areas of the country with different vegetation types.

Precipitation also contributed to the model, and this is a known factor in the distribution of African ungulates since it is related to vegetation growth [51]; however, the nature of the influence of this variable in our results is not very clear since the response curve presented a wide variation in areas of high precipitation (Figure 2). This may be a consequence of high variability of precipitations in areas with no recorded presence of bushpigs, or it may indicate that this variable is capturing information from other correlated variables, either the NDVI, which was at the limit of the correlation threshold that we established (*r* = 0.7) (Supplementary Figure 2) or with other variables not included in our model whose effect could be masked by the precipitation.

There is limited information in the literature from the African-Malagasy region on the factors influencing the presence of bushpigs. Bushpigs are crop raiders that cause frequent problems for local agriculturalists and could be attracted to farming areas due to increased food availability [22, 46]. Previous studies indicated that bushpigs may be frequently found (27%–60%) around villages in certain areas of Madagascar [3]. However, crop and human variables were not found to be major contributors to the probability of bushpig occurrences in our predictive model. It must be kept in mind that the layers of crops used in this study are estimates obtained through modeling [29]. They provide enough spatial resolution to use this information in predictive models, but they could be inaccurate at a local level and prone to offer outdated information. It also must be noted that suitability is not necessarily correlated with abundance, especially when models are driven by only climatic variables [52]. Other variables, e.g., related with food availability, could lead to higher numbers of animals despite the fact that the suitability score may not be the highest. In this regard, crops could still be modulators of the behavior of bushpig populations in areas where these animals are already present by favoring their local movement from adjacent habitats and by increasing their density due to the higher availability of resources. In the case of Madagascar, this would explain the areas of high suitability for bushpigs located in zone III, which overlaps with the high density of domestic pigs. In this regard, the presence of bushpigs around pig farms from this zone has already been reported [3]. This high risk of contact is not only limited to the areas where high densities of domestic and wild swine overlap but also in adjacent areas. For example, in Uganda, bushpigs may have a greater occurrence during the day in areas with denser vegetation and at night venture into more open areas to forage including agricultural plots [19].

The information obtained in this study could be useful for the management of diseases shared between bushpigs and domestic pigs and contribute to a better understanding of the epidemiologic role of bushpigs. The elusive behavior and nocturnal habits of bushpigs [53] can make direct interactions with domestic pigs difficult, hindering disease transmission. Despite this, there are several reports of contacts between bushpigs and domestic pigs in Madagascar and even interbreeding in diverse areas, although not scientifically proven to date [8, 23, 53]. In addition, different practices may favor indirect contact between bushpigs and domestic pigs, including feeding pigs with leftover food wastes, remains of slaughtered animals, inappropriate disposal of dead animals, and, in general, the lack of biosecurity and sanitary measures [21]. Furthermore, bushpigs may ingest contaminated pig carcasses during nocturnal visits to farming areas. Bushpigs are also a favored game species in certain areas [3, 22], and hunting can favor the transmission of disease to domestic pigs when pigs are fed portions of bushpig carcasses [54] and when bushpig hunters or vendors also raise pigs and keep live or butchered bushpigs in close proximity to pig farms.

Therefore, to assess interspecies transmission, the definition of risk areas of contact between domestic pigs and bushpigs is critical. For example, ASF is a relevant endemic disease in the country, and bushpigs are suspected reservoirs [8, 55, 56], but bushpigs have shown a low capacity to become infected, and information about bushpig-domestic pig transmission in field conditions is extremely limited [11, 57]. Antibodies against ASF virus in bushpigs have not been detected in two previous investigations including those on 27 and 26 individuals carried out in the country [58, 59], but studies on the prevalence of ASFV in bushpigs are difficult to conduct and still very local [8]. Moreover, ASF is also transmitted by ticks of the *Ornithodoros* spp., and species of this genus (*Ornithodoros porcinus*) have been found infected in the country [60]. Thus, identifying overlapping areas between potential reservoirs may aid to define surveillance schemes focused on areas with a higher risk of contact between reservoirs and therefore increase the sensitivity of the detection of potential events of interspecies transmission. This would give a better knowledge of the real involvement of bushpigs in the epidemiology of the disease in the country.

This study includes one of the largest assembled datasets of bushpig occurrences on Madagascar and covers a 30-year period. The distributional range of this species could have varied during this period due to different factors: human activity, changes in the suitability of different areas, etc., thus affecting our model. However, we believe that these potential factors have little influence on our results. Instead, the continued presence of bushpigs at certain sites during the three decades (Supplementary Figure 1) suggests the high suitability of these areas for this species, and our environmental variable extrapolations are assumed to be reliable. Likewise, domestic pig data layers were obtained by interpolation, and there may be some problems in accuracy at spatial or temporal levels. However, these layers seem consistent with the district-level information available from administrative sources [61]. However, our model can still be improved. Seasonal changes may have an effect on the distribution of bushpigs and could be modeled in further studies. In terms of risk, modeling abundance would be especially valuable, as density may be more important than mere presence. Moreover, layers of crops and human activities had little impact despite the fact that they could attract wild swine [3]. It cannot be ruled out that low precision at a local scale could misrepresent their influence and predictive value. Different approaches using other methodologies, such as multicriterion decision analysis could also explore this issue from another perspective [62], and it would be valuable to compare different approaches to increase the accuracy of estimates.

## 5. Conclusions

Our results are insightful to design further epidemiological studies on the role of these animals in the epidemiology of diseases such as ASF and help design more cost-effective, risk-based surveillance programs. For example, bushpig sampling should consider areas in which interspecies contacts are more likely to maximize the probability of detection. Monitoring or surveillance systems in defined high-risk areas may give better results to detect sporadic events of transmission that could contribute to the understanding of the risk of transmission at the pig-bushpig interfaces and the design of better mitigation strategies if necessary. This information can also be used for the diverse shared diseases between bushpigs and domestic pigs present in the country in addition to ASF, such as classical swine fever, cysticercosis, trichinellosis, or Hepatitis E [22]. In addition, the methodology implemented here could also be extrapolated to other African countries, contributing to the evaluation of the potential risks of interspecies transmission of pathogens.

## Figures and Tables

**Table 1:** Estimates of the relative contribution of variables included in the ecological niche model.

Variable	Percent contribution	Permutation importance
Normalized difference vegetation index (NDVI)	51.3	66.9
Percentage of land covered by trees	17.6	0.4
Annual-averaged monthly precipitation	12.6	19.0
Evapotranspiration	9.9	3.8
Minimum temperature in the coldest month	5.0	6.1
Maximum temperature in the warmest month	3.6	3.8

## Data Availability

The data supporting the findings of this study are available from the corresponding author upon request.

## Supplementary Materials

Supplementary Materials Supplementary Figure 1: Spatial and temporal distribution of the total number of observations of bushpigs originally gathered (*n* = 206, left) and of the observations included in the ecological niche model (*n* = 83, right) after correction for pseudoreplication. Supplementary Figure 2: The Spearman's correlation matrix of the 36 environmental variables selected for the study. Supplementary Figure 3: The map of the density of pigs (no. of heads/km^2^) in Madagascar. Supplementary Figure 4: Variation in the repetition of the MaxEnt analysis resampling different presence points: (a) the final model presented in the study and used as a reference; (b) the map of the average absolute difference in the predicted output between each of the 1,000 analyses and the reference map; and (c) maximum, 95th percentile, and median of the variation (in %) from the reference map for each of the 1,000 iterations. Supplementary Table 1: Variables considered for the ecological niche model and source. Supplementary Code 1: Example of an R code to correct for pseudoreplication.
