# Supplementary material for: A Maximum Entropy Species Distribution Model to Estimate the Distribution of Bushpigs on Madagascar and Its Implications for African Swine Fever
Source: Transbound Emerg Dis. 2023 Feb 28;2023:7976252. doi: 10.1155/2023/7976252 (PMC12017021; doi:10.1155/2023/7976252)
Supplement: Supplementary Materials — Supplementary Figure 1: Spatial and temporal distribution of the total number of observations of bushpigs originally gathered (n = 206, left) and of the observations included in the ecological niche model (n = 83, right) after correction for pseudoreplication. Supplementary Figure 2: The Spearman's correlation matrix of the 36 environmental variables selected for the study. Supplementary Figure 3: The map of the density of pigs (no. of heads/km2) in Madagascar. Supplementary Figure 4: Variation in the repetition of the MaxEnt analysis resampling different presence points: (a) the final model presented in the study and used as a reference; (b) the map of the average absolute difference in the predicted output between each of the 1,000 analyses and the reference map; and (c) maximum, 95th percentile, and median of the variation (in %) from the reference map for each of the 1,000 iterations. Supplementary Table 1: Variables considered for the ecological niche model and source. Supplementary Code 1: Example of an R code to correct for pseudoreplication. [file 7976252.f1.zip › Supplementary Material R.docx]

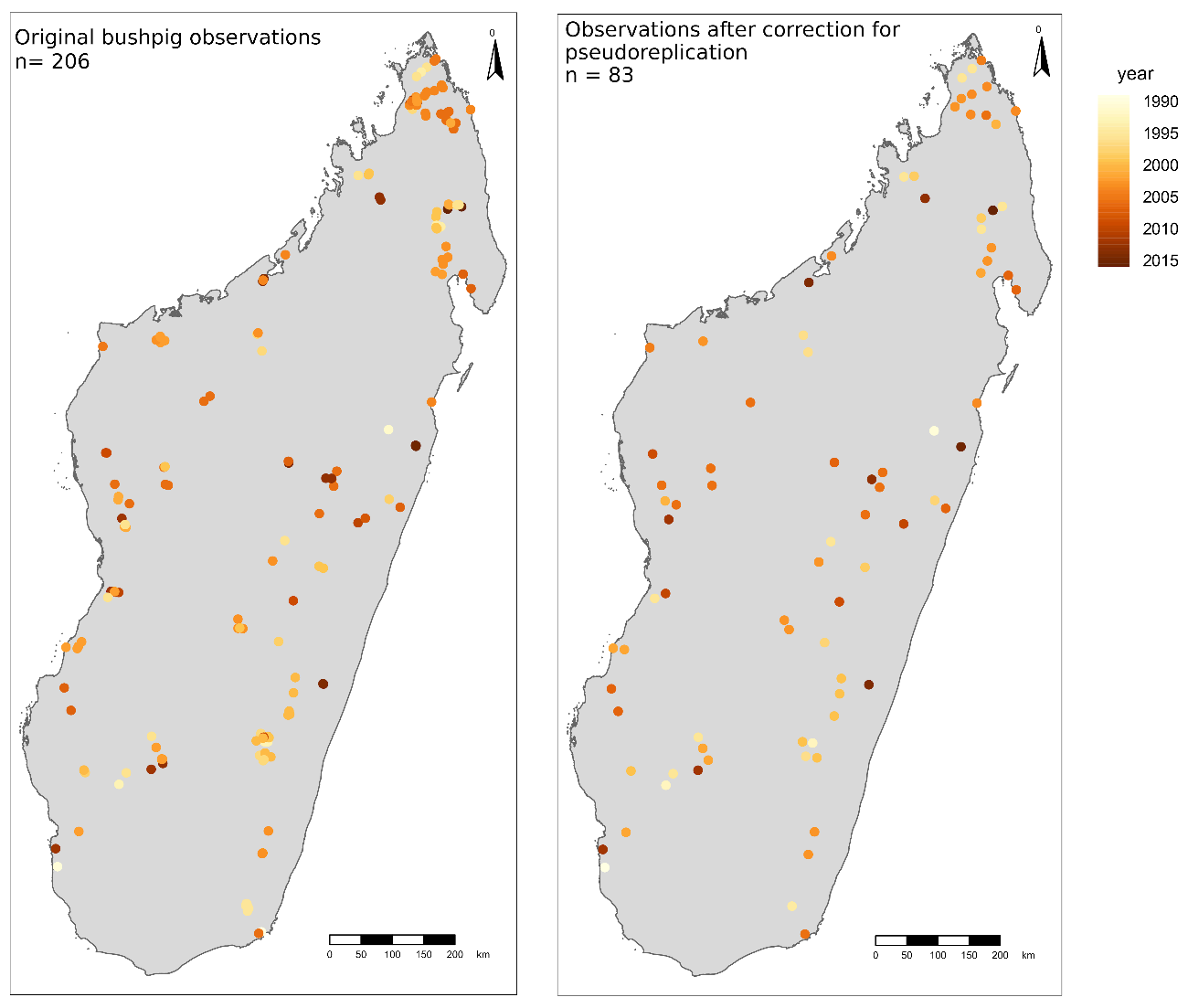


**Supplementary Figure 1.** Spatial and temporal distribution of the number total of observations of bushpigs originally gathered (n = 206, left) and of the observations included in the ecological niche model (n = 83, right) after correction for pseudoreplication.


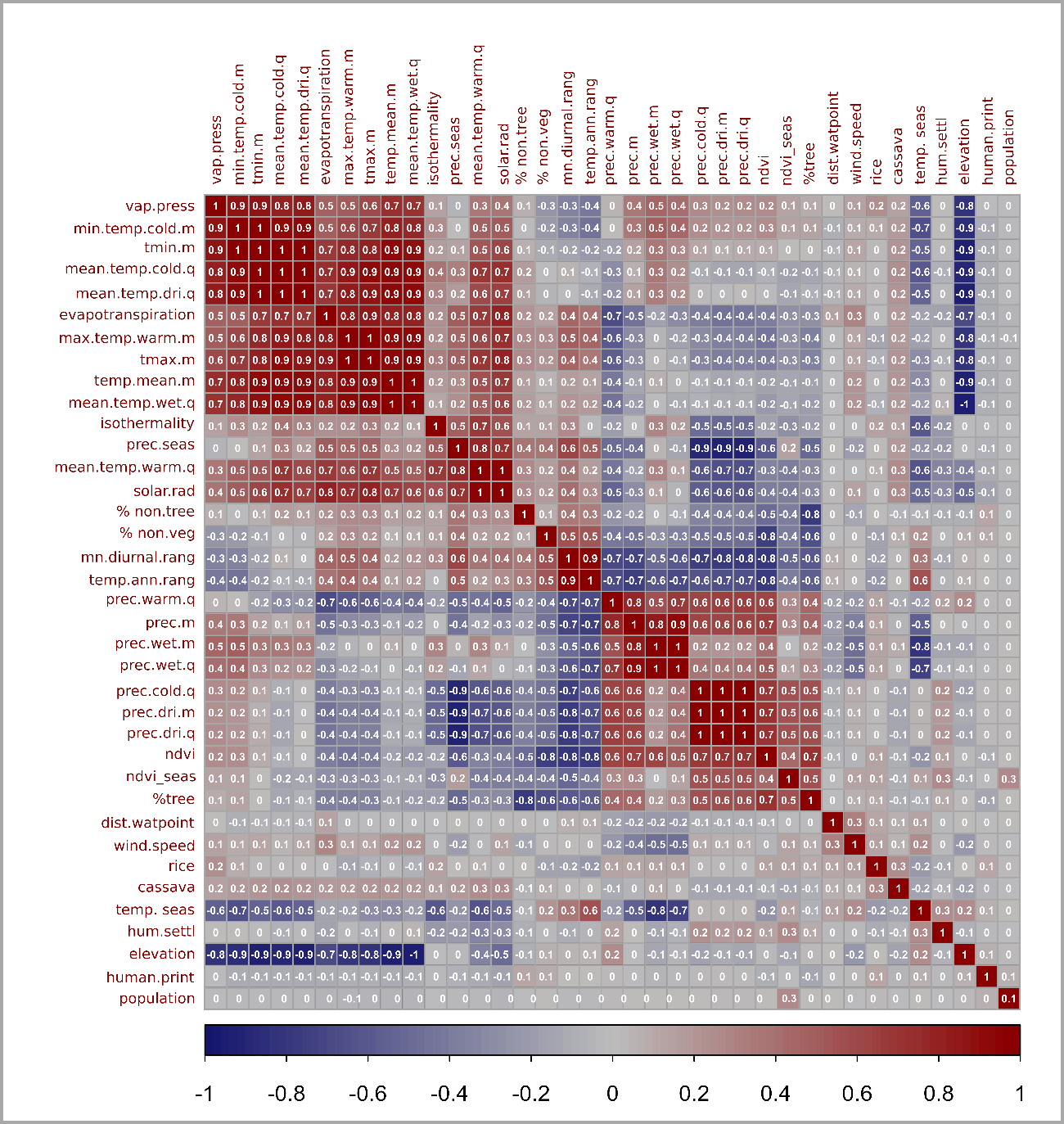


**Supplementary Figure 2**. Spearman’s correlation matrix of the 36 environmental variables selected for the study.

Abbreviations: %*nontree*: percentage of land area occupied by non-tree vegetation cover; %*nonveg*: percentage of land area occupied by bare soil; %*tree*: percentage of land area occupied by tree cover; *cassava*: cassava production; *dist*.*watpoint*: distance to water points; *elevation*; *evapotranspiration*; *human*.*print*: human footprint; *hum*.*settl*: distance to human settlements (cities/towns); *isothermality*; *max.temp.warm.m*: maximum temperature of warmest month; *mean.temp.cold.q*: mean temperature of coldest quarter; *mean.temp.dri.q*: mean temperature of driest quarter; *mean.temp.warm.q*: mean temperature of warmest quarter; *mean.temp.wet.q*: mean temperature of wettest quarter; *min.temp.cold.m*: minimum temperature of coldest month; *mn.diurnal.rang*: mean diurnal range; *ndvi*: normalized difference vegetation index; *ndvi_seas*: normalized difference vegetation index seasonality; *population*: human population; *prec.cold.q*: precipitation of coldest quarter; *prec.dri.q*: precipitation of driest quarter; *prec.dri.m*: precipitation of driest month; *prec.m*: annual average of monthly precipitation; *prec.seas*: precipitation seasonality; *prec.warm.q*: precipitation of warmest quarter; *prec.wet.q*: precipitation of wettest quarter; *prec.wet.m*: precipitation of wettest month; *rice*: rice production; *solar.rad*: solar radiation; *temp.ann.rang*: temperature annual range*; temp.mean.m*: annual average of monthly temperature; *temp.seas*: temperature seasonality; *tmax.m*: annual average of maximum monthly temperature; *tmin.m*: annual average of minimum monthly temperature; *vap.press*: vapor pressure; *wind.speed*


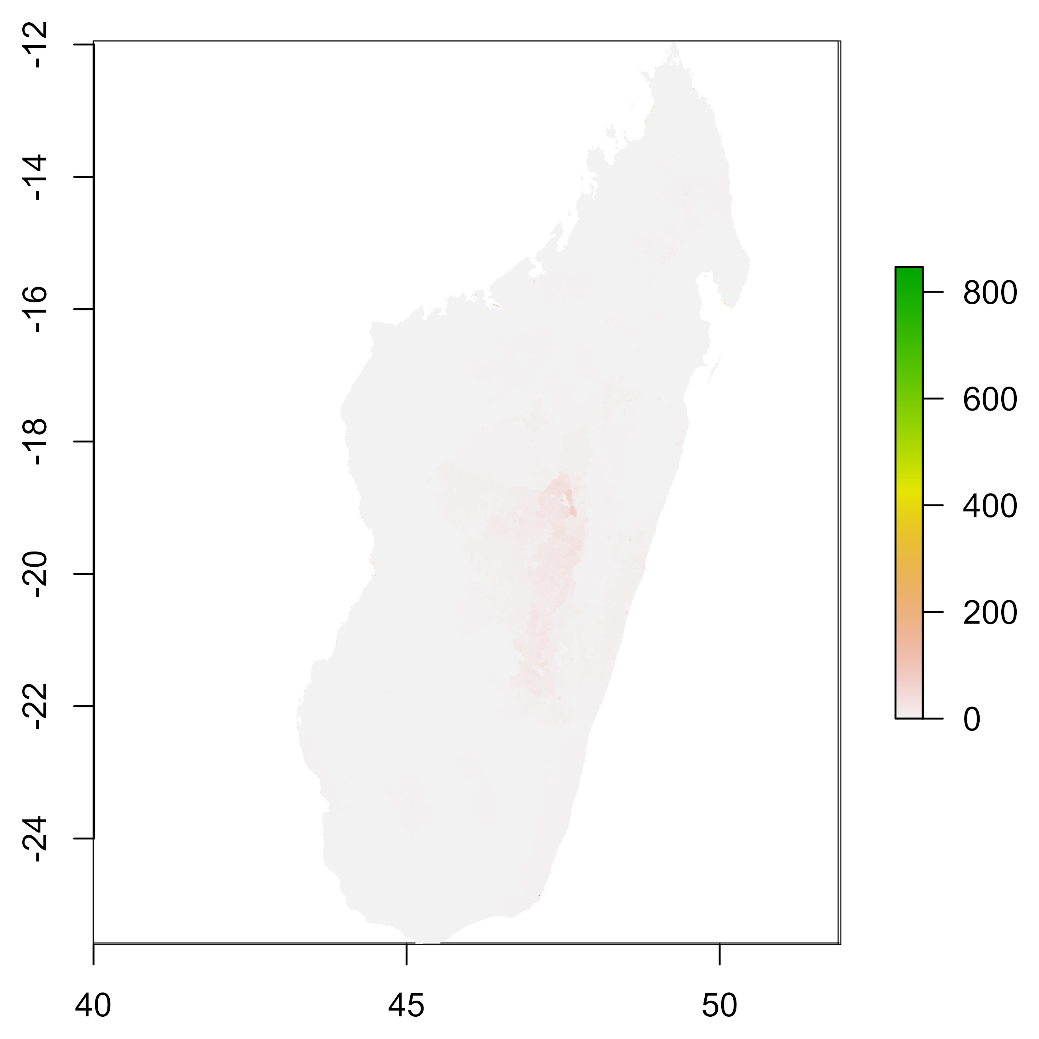


**Supplementary Figure 3**. Map of the density of pigs (No of heads/km^2^) in Madagascar. (Retrieved from: <https://livestock.geo-wiki.org> [accessed on 20/07/2022])


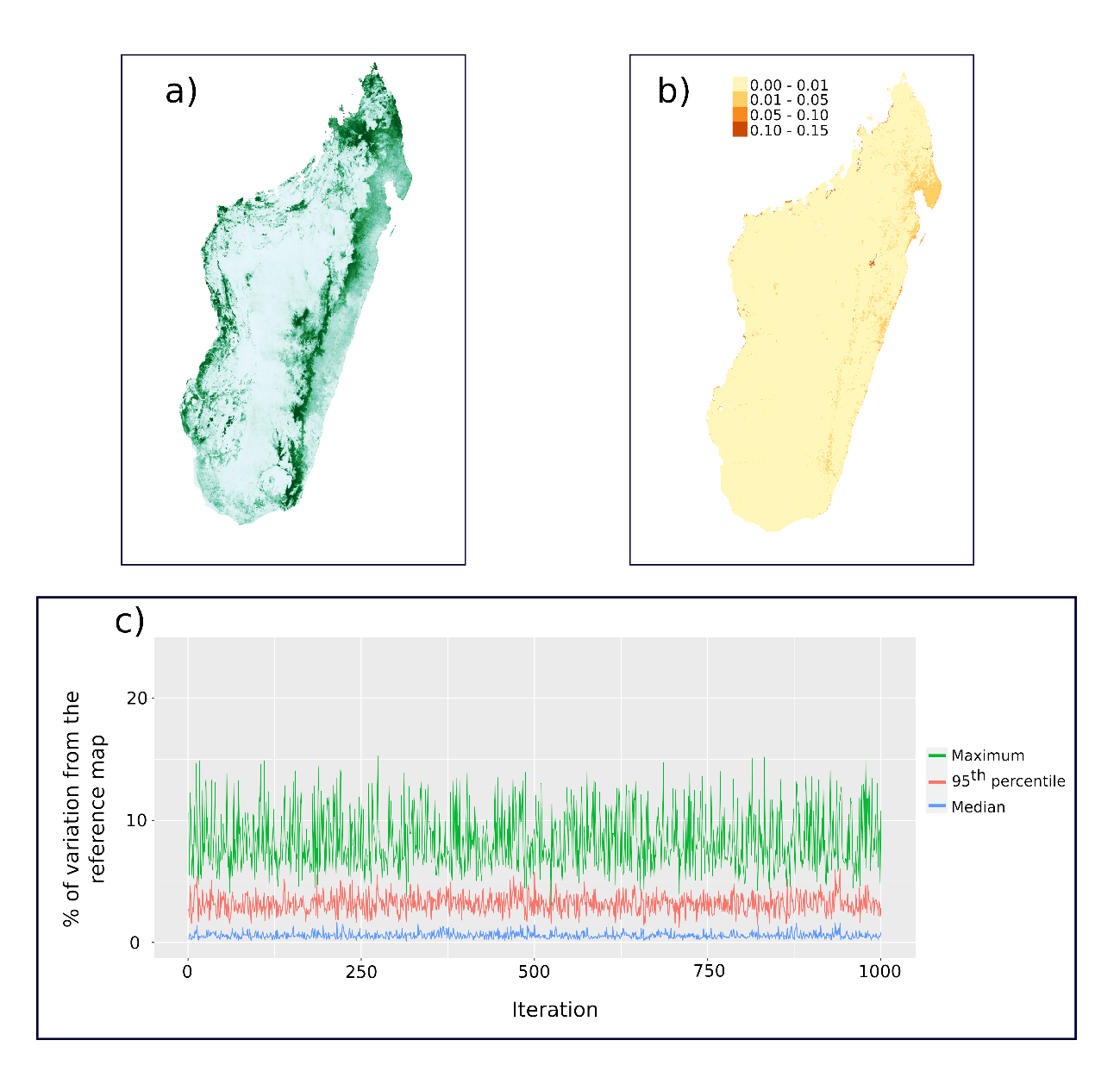


**Supplementary Figure 4.** Variation in the repetition of the MaxEnt analysis resampling different presence points: a) final model presented in the study and used as reference; b) map of average absolute difference in the predicted output between each of 1,000 analysis and the reference map; c) Maximum, 95th percentile and median of the variation (in %) from the reference map for each of 1,000 iterations.

**Supplementary Table 1**. Variables considered for the ecological niche model and source.

| Variable | Group | Source |
| --- | --- | --- |
| Annual average of monthly temperature | Climatic | Calculated from data retrieved from: worldclim.org |
| Annual average of minimum monthly temperature |  |  |
| Annual average of maximum monthly temperature |  |  |
| Annual average of monthly precipitation |  |  |
| Annual average of monthly wind speed |  |  |
| Annual average of monthly solar radiation |  |  |
| Annual average of monthly water vapor pressure |  |  |
| Mean Diurnal Range |  | worldclim.org |
| Isothermality |  |  |
| Temperature Seasonality |  |  |
| Maximum temperature of warmest month |  |  |
| Minimum temperature of coldest month |  |  |
| Temperature annual range |  |  |
| Mean temperature of wettest quarter |  |  |
| Mean temperature of driest quarter |  |  |
| Mean temperature of warmest quarter |  |  |
| Mean temperature of coldest quarter |  |  |
| Precipitation of wettest month |  |  |
| Precipitation of driest month |  |  |
| Precipitation seasonality |  |  |
| Precipitation of wettest quarter |  |  |
| Precipitation of driest quarter |  |  |
| Precipitation of warmest quarter |  |  |
| Precipitation of coldest quarter |  |  |
| Normalized difference vegetation index (NDVI) | Vegetation | Moderate Resolution Imaging Spectroradiometer (MODIS)  urs.earthdata.nasa.gov |
| Evapotranspiration |  |  |
| Percentage of land area occupied by tree cover |  |  |
| Percentage of land area occupied by non-tree vegetation cover |  |  |
| Percentage of land area occupied by bare soil |  |  |
| NDVI seasonality |  | Calculated from NDVI as the coefficient of variation of the average monthly NDVI rasters |
| Elevation | Geographic | Retrieved from:  wordclim.org |
| Distance to water points |  | Calculated from data retrieved from: openstreetmap.org |
| Distance to human settlements |  |  |
| Rice production | Agricultural/human | International Food Policy Research Institute. Retrieved from: dataverse.harvard.edu/dataverse/harvestchoice |
| Cassava production |  |  |
| Human population |  | Calculated from data retrieved from:  worldpop.org |
| Human footprint |  | NASA Socioeconomic Data and Applications Center. Retrieved from:  sedac.ciesin.columbia.edu/data/set/wildareas-v3-2009-human-footprint |
